# Patient Safety and Satisfaction With Fully Remote Management of Radiation Oncology Care

**DOI:** 10.1001/jamanetworkopen.2024.16570

**Published:** 2024-06-12

**Authors:** John J. Cuaron, Sean McBride, Fumiko Chino, Dhwani Parikh, Marisa Kollmeier, Gerri Pastrana, Keri Wagner, Andrew Tamas, Daniel Gomez

**Affiliations:** 1 Department of Radiation Oncology, Memorial Sloan Kettering Cancer Center, New York, New York

## Abstract

**Importance:**

Patients of Memorial Sloan Kettering Cancer Center in New York, New York, are now offered a choice of either in-person or remote telehealth visits for radiation oncology care. However, safety and satisfaction among patients receiving treatment with fully remote physician management is unclear.

**Objective:**

To analyze patient safety and satisfaction, financial implications, and environmental consequences associated with fully remote management among a cohort of patients treated with radiotherapy.

**Design, Setting, and Participants:**

This single-institution retrospective cohort study was performed at Memorial Sloan Kettering Cancer Center, with patients treated with radiation who opted for fully remote management between October 1, 2020, and October 31, 2022. Data on patient safety events were prospectively collected with an in-house quality improvement reporting system. Patient satisfaction surveys were distributed electronically before, during, and after treatment. Patient transportation costs and environmental consequences were estimated based on differences in travel distance. Data analysis was performed from March 14 through September 19, 2023.

**Exposure:**

Radiotherapy with fully remote physician management.

**Main Outcomes and Measures:**

Satisfaction rates among patients opting for fully remote management were analyzed via surveys administered electronically after visits with clinicians. Patient safety events, defined as staff-reported actual events and near misses that had the potential to affect patient care, were reviewed. Rates and types of safety events were analyzed and compared with patients treated by onsite clinicians. Distances between patient home zip codes and treatment site locations were compared with estimated cost savings and decreased emissions.

**Results:**

This study included 2817 patients who received radiation oncology care with fully remote physician management. The median age of patients was 65 (range, 9-99) years, and more than half were men (1467 [52.1%]). Of the 764 safety events reported, 763 (99.9%) did not reach patients or caused no harm to patients. Nearly all survey respondents (451 [97.6%]) rated patient satisfaction as good to very good across all domains. For treatment with fully remote physician management, out-of-pocket cost savings totaled $612 912.71 ($466.45 per patient) and decreased carbon dioxide emissions by 174 metric tons.

**Conclusions and Relevance:**

In this study, radiation oncology care provided by fully remote clinicians was safe and feasible, with no serious patient events. High patient satisfaction, substantial cost savings, and decreased environmental consequences were observed. These findings support the continuation of a fully remote management option for select patients in the post–COVID-19 era.

## Key Points

**Question:**

How do patients receiving radiation oncology care with fully remote physician management rate their satisfaction with this option, and what are the associated safety events, financial implications, and environmental consequences?

**Findings:**

In this cohort study of 2817 patients, more than 99% of safety events did not reach patients or caused no harm to patients and 98% of patient ratings of satisfaction with fully remote management were good to very good. Out-of-pocket cost savings associated with fully remote management totaled approximately $612 913 ($466 per patient), and estimated carbon dioxide emissions decreased by 174 metric tons.

**Meaning:**

These findings suggest that a fully remote management option for properly selected patients receiving radiotherapy is safe and may preserve patient and clinician flexibility, expand access, create less financial toxicity, and reduce the carbon footprint.

## Teaser

This cohort study evaluates patient safety and satisfaction, financial implications, and environmental consequences associated with fully remote physician management of radiation oncology care.

## Introduction

The COVID-19 pandemic created an unprecedented need to quickly adapt oncology practice to deliver effective care while simultaneously protecting vulnerable populations (eg, patients with cancer) from exposure to SARS-CoV-2 infection. The rapid expansion of telehealth practice allowed access to clinicians while minimizing SARS-CoV-2 infection risk.^1^ Prior research shows that most patients view telehealth favorably with substantial benefits, allowing for flexibility and enhancing clinician ability to provide patient-centered care^2,3^ without decreases in key quality-of-care indexes.^4^ Maximized expansion of telehealth offerings has been posited to be an important effort to advance health equity via enhanced access and reduced financial toxicity,^5^ given travel cost savings^6^ and improved ability to receive specialty services.^2,7,8^ Remote monitoring of clinical trials may also be feasible and safe,^9^ allowing more diverse populations to enroll in clinical research.^10^

In a previous study, we described excellent patient satisfaction and treatment confidence among those receiving radiation during a period of mandated remote care^11^ in the early COVID-19 pandemic. Upon restoration of in-person visits, many institutions decreased service offerings delivered remotely, despite durable positive patient experiences with telemedicine even later in the pandemic.^3^ Because radiation as a daily treatment has a high travel burden,^12,13^ the ability to deliver radiation closer to home has clear patient-centered benefits. Alternatively, treatment at a quaternary referral academic cancer center has potential outcome advantages, including greater use of a shorter treatment course and advanced technologies^14,15,16^ that can improve personalized and precision oncology care^17,18^ and potentially increase survival.^19^

To expand patient access, meet the high demand for care closer to home, and preserve continuity of care, our institution established a completely remote care model using an expanded telehealth infrastructure. This model allowed complete care delivery, including the initial consultation, radiation treatment plan design and approval, and on-treatment management, to be delivered remotely throughout the entire regional network, independent of the treating physician’s physical location. This study analyzes patient safety and satisfaction, financial implications, and environmental consequences of the fully remote management model among a cohort of patients with cancer treated with radiotherapy.

## Methods

We conducted this retrospective observational cohort study of patients treated with radiation therapy via complete remote physician management between October 1, 2020, and October 31, 2022. The study was completed as part of an approved Memorial Sloan Kettering Cancer Center Institutional Review Board protocol. Patients provided written informed consent. We followed the Strengthening the Reporting of Observational Studies in Epidemiology (STROBE) reporting guideline.

### Patient Population

**Figure 1.**
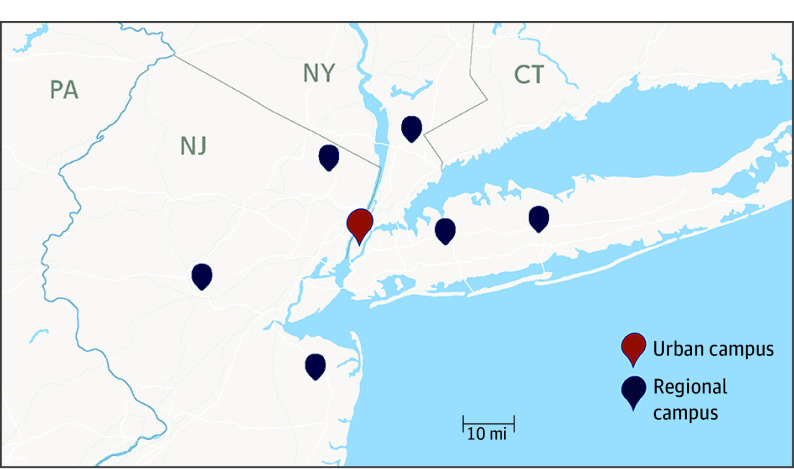
Memorial Sloan Kettering Cancer Center Campus Map The regional map shows the locations of the urban campus in Manhattan, New York, and the 6 regional sites in Westchester County, New York (n = 1), Long Island, New York (n = 2), and New Jersey (n = 3). The coordinates are as follows: urban campus (40.77369179310323, −73.95488308285093); New Jersey campus 1 (41.077452366674244, −74.07134020458915), campus 2 (40.371715860754755, −74.14679252645489), and campus 3 (40.63989328702856, −74.58354811942598); and New York campus 1 (41.02830612378613, −73.73355268110447), campus 2 (40.71988582716729, −73.59127861076811), and campus 3 (40.80979966664221, −73.2917929777375).

In recent years, a higher percentage of patients at our institution elected to initiate radiation therapy in our community-based regional network sites in New York and New Jersey outside of Manhattan, New York (54.4% in the regional network in 2019 vs 59.3% in 2020, 63.1% in 2021, and 63.1% in 2022). This shift created a strain on resources and staff at the regional centers and disruptions in care due to the need for patient transitions between clinicians at different locations. To address these challenges, the Remote Management Radiation Treatment Program was designed as a phased expansion at our 6 regional clinic sites (Figure 1). Thirty radiation oncology faculty with full organ site–specific expertise and experience practice at the regional sites. Each site has full computed tomography and positron emission tomography simulation capability and at least 3 linear accelerators with full treatment modality capability. Additionally, 3 of the 6 regional campuses are equipped with high dose-rate brachytherapy for the delivery of intravaginal radiotherapy. Patients undergoing radiation planning (simulation) at one campus may easily receive care at any center within the network or at the urban center. Each site also has an onsite radiation oncologist (ie, “doctor of the day”) available to evaluate and manage any urgent patient treatment–related issues.

The remote management program was initiated in October 2020. Patients were determined as eligible if they met the following criteria: (1) the availability for new-visit consultations with preferred onsite clinicians was longer than 7 days, or patients expressed preference for treatment at a facility different from the oncologist’s primary location; (2) the treatment plan included a limited number of radiation treatments (fractions ≤5) without chemotherapy; and (3) clinicians determined that patients had appropriate performance status and electronic literacy for telehealth. After an initial pilot period, the remote management program was expanded in December 2020 to a larger cohort of patients receiving definitive treatment or a higher number of radiation treatments. Remote physician management started with either an initial in-office or telehealth consultation, and then patients were scheduled to receive daily radiation treatment at a regional network site closest to their home. The treating physician prescribed treatment, designed the treatment plan, and conducted weekly management visits with the patient via telehealth (requiring both an audio component and a visual component).

At the outset of the remote management program, weekly huddles were led by physician leaders and included radiation therapists, nursing staff, physicians, and clinic supervisors. At each huddle, the following metrics were reviewed: (1) number of patients, (2) diagnosis, (3) wait times to treatment planning procedure (simulation), (4) regional campus treatment end times, (5) documentation errors, and (6) serious patient care events. Through huddle discussions, a framework for triage and accountability was developed and implemented in later stages of the program. Onsite nursing teams and the doctor of the day addressed patient reports of new side effects or symptoms, whereas the remote physician team responded to nonurgent patient-directed questions regarding treatment plans, medications, outcomes, or follow-up.

### Statistical Analysis

Statistical analysis was performed using R software, version 4.3.1 (R Project for Statistical Computing); *P* < .05 was considered statistically significant. Data analysis was performed from March 14 through September 19, 2023.

#### Patient Safety

To monitor patient safety, events were documented with our in-house event reporting system, RISQ (Reporting to Improve Safety and Quality). The RISQ system is designed to allow staff members to report both actual events and events that have the potential to cause harm but are caught and corrected before they reach the patient. At our institution, RISQ has a high level of use and is the main mechanism for collecting, analyzing, and acting on patient safety events and workflow process improvement. The types of events collected include patient condition and decline, provider communication, equipment issues, and nonpatient-facing treatment planning issues, among others. Events are graded based on their severity as follows: 0, did not reach patient; 1, no harm; 2, temporary or minor harm; 3, permanent or significant harm; or 4, death. Safety events were discussed in the aforementioned weekly huddles. The 2-sided χ^2^ test was used to compare the number of events per patient who received remotely managed care vs patients who received nonremotely managed care in previous years.

#### Patient Satisfaction

The instruments and methods used to assess patient satisfaction have been described previously.^11^ Patient satisfaction surveys were distributed electronically and administered via a text message link sent to the patient’s telephone number of record after initial consultations and during and after treatments. The survey questions are provided in the eAppendix in Supplement 1.

To estimate environmental consequences and decreases in patient travel costs associated with complete remote physician management, travel distances between regionally treated patient home zip codes, the urban campus, and the regional campus of treatment were calculated using the Haversine formula^20^ based on latitude and longitude as ascertained by Google Maps (Alphabet Inc). Differences in travel costs were estimated based on the 2021 federal standard mileage rate of 56 cents per mile,^21^ multiplied by 2 to account for round trips, multiplied by the number of treatment visits for each patient, and included differences in parking costs. Carbon dioxide emission estimates were based on an average automobile emission of 400 g per mile.^22^

## Results

### Patient Characteristics

This study included 2817 patients who received radiation oncology treatment with remote physician management. Patient characteristics are shown in Table 1. The median age of patients was 65 (range, 9-99) years; 1467 (52.1%) were men and 1350 (47.9%) were women. The most common disease sites were bone metastases (538 [19.1%]), nonbone metastases (511 [18.1%]), prostate or genitourinary cancer (439 [15.6%]), and breast cancer (295 [10.5%]). Overall, 1094 patients (38.9%) who received remotely managed care had metastatic disease. The distribution of diagnoses among patients treated without remote management during the same period (n = 22 178) was similar, except the previous percentage of breast cancer was higher (4506 [20.3%]).

**Table 1.** Patient Characteristics

Characteristic	Values^a^
Age at treatment, median (range), y	65 (9-99)
Sex	
Male	1467 (52.1)
Female	1350 (47.9)
Metastatic disease	1094 (38.9)
Disease site	
Brain, spine, or meninges	76 (2.7)
Breast	295 (10.5)
Gastrointestinal	144 (5.1)
Gynecologic	33 (1.2)
Head and neck, skin, or eye	174 (6.2)
Hepatobiliary	127 (4.5)
Lung	156 (5.5)
Lymphoma	92 (3.3)
Bone metastasis	538 (19.1)
Nonbone metastasis	511 (18.1)
Prostate or genitourinary	439 (15.6)
Sarcoma	147 (5.2)
Unclassified or not otherwise specified	85 (3.0)

^a^ Unless indicated otherwise, values are presented as the No. (%) of patients.

**Figure 2.**
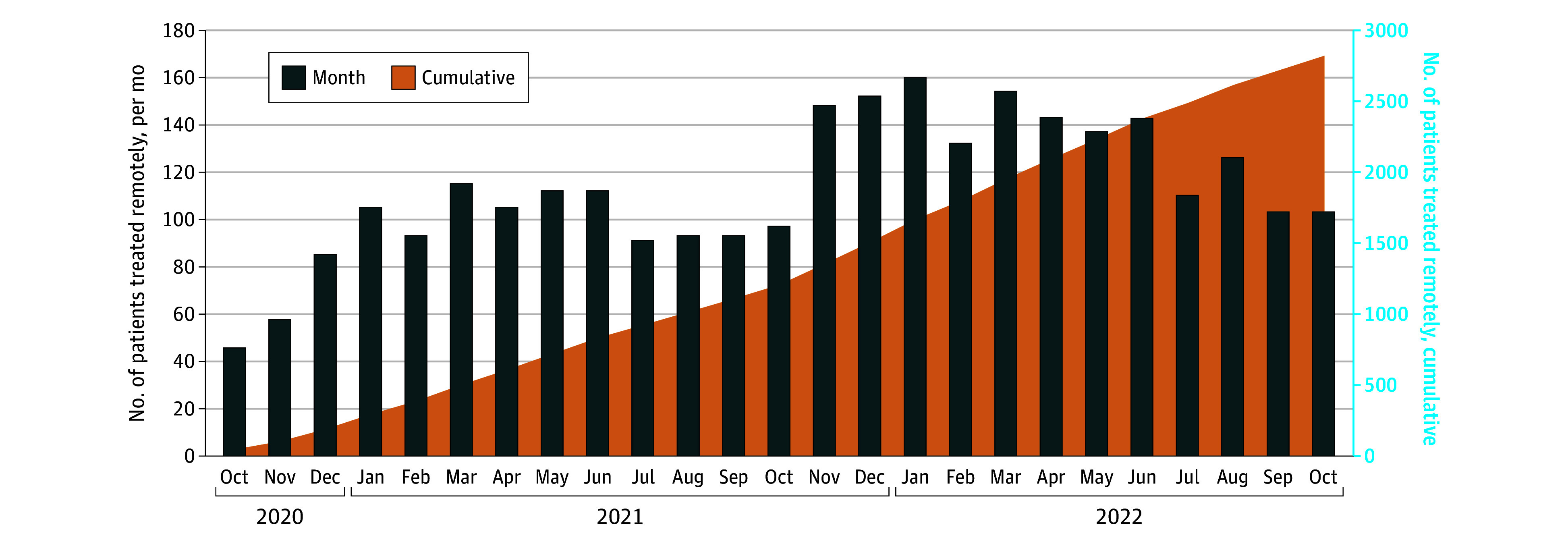
Monthly and Cumulative Numbers of Patients Receiving Radiation Oncology Care With Fully Remote Management

At the end of the study period, the number of patients treated in the department who received remotely managed care ranged from 100 to 160 per month. This volume represented approximately 10% to 15% of patients treated in the department at large and between 10% and 35% of patients receiving treatment at each of the regional network sites (Figure 2).

### Adverse Patient Safety Events

A total of 764 adverse patient safety events were reported for patients treated with remote management during the study period, at a rate of 0.27 events per patient per year. This rate was higher than that in 2019 (before initiation of the Remote Management Radiation Treatment Program), which was 0.15 events per patient per year (*P* < .05). Of the 764 safety events reported, 763 (99.9%) did not reach patients (grade 0, 308 [40.3%]) or caused no harm to patients (grade 1, 455 [59.4%]). One patient experienced a seizure while undergoing treatment, which required activation of emergency services; this was a grade 2 event (temporary or minor harm) and was not attributed to remote management. There were no grade 3 or 4 events.

Events were classified in categories based on the nature of the event. The categories were as follows: treatment planning issues (relating to the iterative process of generating an optimal radiation treatment delivery program) (323 [42.2%]), treatment planning procedure (simulation) or treatment delivery delay (90 [11.8%]), scheduling errors (82 [10.7%]), physician order entry (72 [9.4%]), treatment delivery (51 [6.7%]), treatment consent collection (50 [6.5%]), radiation therapy prescription issues (38 [5.0%]), equipment issues (23 [3.0%]), handoff or sign out issues (12 [1.6%]), patient condition (12 [1.6%]), and patient education or preparation (12 [1.6%]). The distribution of events is shown in Table 2.

**Table 2.** Adverse Patient Safety Events

Safety event category	No. (%) of patients
Treatment consent collection	50 (6.5)
Equipment issue	23 (3.0)
Handoff or sign-out issue	12 (1.6)
Physician order entry	72 (9.4)
Patient condition	12 (1.6)
Patient education or preparation	12 (1.6)
Radiation therapy prescription issue	38 (5.0)
Scheduling error	82 (10.7)
Simulation or treatment delay	90 (11.8)
Treatment delivery delay	51 (6.7)
Treatment planning issue	323 (42.2)
Total	765 (100)

### Patient Experience and Satisfaction

**Figure 3.**
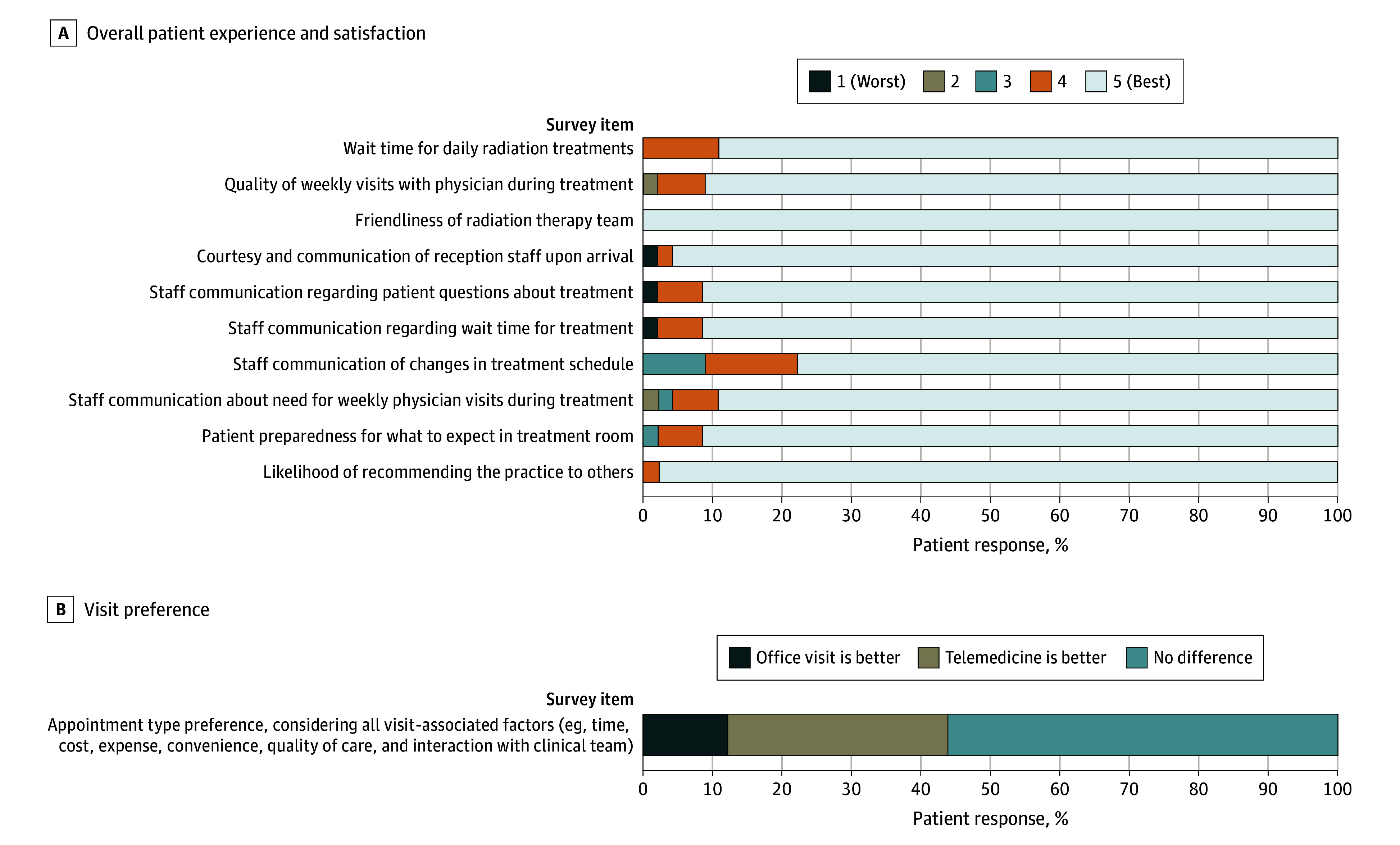
Patient Experience and Satisfaction With the Fully Remote Management Program Patient responses to satisfaction survey questions (eAppendix in Supplement 1) were rated on a scale from 1 (worst) to 5 (best).

The survey response rate was 31.0% (n = 873). Overall patient satisfaction remained high during the entirety of the surveyed period, with nearly all survey responses (451 [97.6%]) rating satisfaction as good to very good across all domains. Most respondents (36 [87.8%]) either preferred telehealth or expressed no preference for in-person vs fully remote visits, whereas the remainder (5 [12.2%]) stated a preference for in-person visits (Figure 3).

### Travel Costs and Environmental Consequences

Of the 2817 patients who received fully remote managed care, 1314 (46.6%) were treated in the regional network. Treatment in the network at sites in New York or New Jersey vs at the Manhattan campus saved a total of 434 530 miles of driving distance, with an average of 330.7 miles per patient during radiation treatment. Regional campus treatment was associated with a total savings of $612 912.71 when accounting for fuel and parking costs, with an average of $466.45 per patient. The decreased travel distance was associated with an estimated carbon footprint reduction of 174 metric tons of carbon dioxide.

## Discussion

To our knowledge, this study represents the largest reported cohort to date of patients treated with radiotherapy in a completely remote fashion throughout a large regional radiation oncology network of an academic cancer center. Patient satisfaction with the program was high, and no serious safety issues were reported. Treatment provided at the regional centers substantially lowered patient out-of-pocket travel costs and reduced the carbon footprint compared with the same treatment courses that would have been administered at the Manhattan campus. Importantly, the remote management program preserved access to specialty care and allowed patients to choose to receive care closer to home.

Since the beginning of the COVID-19 pandemic, telehealth practice has been increasingly incorporated into oncology care. Many studies show a high level of patient satisfaction with telemedicine overall^23^ and with radiation oncology care specifically.^24,25,26,27^ However, some variability has been observed by visit type, with generally high satisfaction with new visits and follow-up visits and mixed results for treatment visits.^28,29,30^ The preference for telehealth vs in-person visits also seems to be related to the reason for the visit. For example, data suggest that patients would be generally accepting of telehealth visits after the pandemic but prefer in-person visits for making highly sensitive treatment decisions and establishing personal connections.^31,32^ However, a 2023 randomized clinical trial showed that remote genetic testing without personalized counseling did not increase distress in patients, suggesting that this avenue may be used to improve access to care for certain services.^33^

Our institution responded to the COVID-19 pandemic by first mandating telehealth visits until safety protocols were enacted. Both patient and clinician satisfaction with telehealth visits during the pandemic peak when in-person visits were restricted was preserved.^11,34^ The findings of this study support a continued favorable view of elective virtual treatment visits, with no apparent shift in preference toward either telehealth or in-person visits over time. An important distinction of fully remote management is that telehealth visits comprise only a component of care. The additional features of expanded access through clinician and treatment location flexibility, a robust patient triage system, and reduced out-of-pocket patient costs are notable benefits of the remote management model and distinguish this study from the expanding teleoncology literature.

In addition to program satisfaction, a high level of safety was also maintained for patients in this study who received fully remote care. This finding is consistent with previous reports investigating the safety of other treatment modalities, including prepandemic comparisons of patients in the Townsville TeleOncology Network in Australia, who received chemotherapy at a tertiary urban center vs a rural outpatient clinic supervised by the same physicians via telemedicine. The authors found no differences in rates of serious side effects, hospital admissions, or mortality between groups.^35^ Similar studies demonstrated that safety, efficiency, and outcomes were preserved during the COVID-19 era among patients treated with systemic therapy managed via telemedicine.^36,37^ To our knowledge, this study represents the first investigation of the safety of remote care among a cohort of patients treated with radiotherapy. Although the rate of safety events per patient (ie, 765 total events, 0.27 events per patient per year) seems high, it reflects the culture of safety within our department and highlights the widespread adoption and ease of use of our patient safety reporting system. All but 1 reported event was designated as either “did not reach the patient” or “no harm,” with nearly two-thirds of events surrounding the treatment planning or documentation aspects of treatment delivery, completely removed from direct patient care. The distribution of patient safety events was similar to that of patients in our department who did not receive remote management, indicating no notable physical or treatment delivery-related risks to patient safety from fully remote care. The fact that the rate of events per patient among patients receiving remote management was statistically higher than in previous years likely represents increased vigilance during initiation of the remote management program, which we anticipate will decrease as remote management workflows are improved.

As the COVID-19 pandemic shifts to the endemic phase, the future of telehealth and remote care of patients remains uncertain. We see 2 main potential advantages to continued remote management. First, the flexible provision of in-person and telehealth visits allows for physical evaluation when needed, while limiting the inconvenience, personal expense, and environmental harm of unnecessary office trips. Certain patient populations, particularly those with metastatic disease with a high symptom burden and low performance status, can particularly benefit from the advantages of remote care given the difficulty of physical relocation for in-office visits. The estimated decreases in financial toxicity and environmental consequences observed in this study corroborate similar reports of the favorable effect of transitioning to teleoncology.^38^ These estimates may still underestimate the total financial implications of treatment closer to home, as they do not account for factors such as lost wages and childcare costs (eg, for patients and family members needing to take time from work and arrange childcare to bring patients to appointments) and additional tolls for bridge and tunnel use. Furthermore, as financial toxicity worsens existing health disparities and disproportionately affects the most vulnerable patient populations,^39,40^ decreased out-of-pocket costs with the use of remote management will contribute to improved health equity. Second, offering patients initial visits in one location and treatment and weekly visits in another, all managed by the same physician, allows for increased access to clinicians who may have availability to see patients sooner. As our experience in the remote management delivery model grows and physician and patient satisfaction remain intact, our institution plans to continue offering the remote management option in the postpandemic era, in line with national preferences to retain telemedicine options after the end of the public health emergency declaration in the US.^26,41,42,43,44^

### Limitations

Our study cohort comprised a highly selected group of technologically literate patients from a single institution. Determination of fitness for remote management was left to individual clinicians and was not standardized. Given the sample size, breadth of diagnoses, and study duration, however, this analysis offers useful insights for other institutions considering a remote management program. A formal assessment of technological literacy was not conducted, which may propagate overestimations of patient readiness for telehealth.^45^ Potential future enhancements of our remote management practice include continuous assessment of patient comfort with both telemedicine and the remote management arrangement and expansion of accessibility features for individuals with hearing or visual impairment. We also acknowledge that successful integration of fully remote radiotherapy is highly dependent on robust quality control, careful communication escalation pathways, standardization of treatment approaches and techniques, unified systems for treatment planning and delivery, and a shared electronic medical record, which may not be as developed in other regionally based facilities and may be challenging to implement across institutions. Although our department’s culture of safety drives a high usage of event reporting for quality improvement, collection of safety events relies on voluntary reporting by staff members, and some safety events may have gone underreported or unreported. Finally, this study was also limited by the lack of patient oncologic outcomes among those treated with remote management. However, given the scarcity of oncologic safety issues encountered during the treatment of patients with remote management, we anticipate no differences in long-term outcomes.

## Conclusions

In this cohort study of a selected group of patients treated with radiotherapy with fully remote physician management, high levels of patient safety and satisfaction were observed. A consistent number of patients treated in our department now opt for remote physician management. These findings suggest that the planned continuation of the program will allow for preservation of patient and clinician flexibility, expanded access, and decreased financial toxicity among properly selected patients.

## Notes

Supplement 1. **eAppendix.** Electronically Administered Patient Satisfaction Survey

Supplement 2.
Data Sharing Statement
