# Supplementary material for: Patient Safety and Satisfaction With Fully Remote Management of Radiation Oncology Care
Source: JAMA Netw Open. 2024 Jun 12;7(6):e2416570. doi: 10.1001/jamanetworkopen.2024.16570 (PMC11170299; doi:10.1001/jamanetworkopen.2024.16570)
Supplement: Supplement 1. — eAppendix. Electronically Administered Patient Satisfaction Survey [file jamanetwopen-e2416570-s001.pdf]

Supplementary Online Content

Cuaron JJ, McBride S, Chino F, et al. Patient safety and satisfaction with fully remote management of radiation oncology care. *JAMA Netw Open*. 2024;7(6):e2416570. doi:10.1001/jamanetworkopen.2024.16570

eAppendix. Electronically Administered Patient Satisfaction Survey

This supplementary material has been provided by the authors to give readers additional information about their work.

© 2024 Cuaron JJ et al. *JAMA Network Open.*

eAppendix. Electronically Administered Patient Satisfaction Survey

Please rank on a scale from 1 to 5, 1 being the worst and 5 being the best:

1. How would you rate the courtesy and communication shown by the clerks and receptionists upon your arrival to the department for simulation?
2. How would you rate the effectiveness of communication by the clerks and receptionists upon your arrival to the department for simulation?
3. How would you rate our communication of wait time for your simulation by the staff?
4. How would you rate our communication regarding any questions you had about your simulation procedure by the staff?
5. How would you rate how well prepared you were for what to expect during simulation?
6. How would you rate the friendliness of the simulation therapist?
7. How would you rate the friendliness of the nurses during your simulation visit?
8. How would you rate the clarity of the explanations the simulation therapist provided you about the simulation procedure?
9. How would you rate the staff's efforts to include you in decisions about your radiation treatment schedule?
10. How likely are you to recommend our practice to others?

Please select your preference:

11. When taking account all of the factors associated with your visit (time, cost, expense, convenience, quality of care, interaction with clinical team, etc), what type of appointment would you have preferred? (Telehealth visit is better, office visit is better, no difference)

© 2024 Cuaron JJ et al. *JAMA Network Open.*
