# Supplementary material for: Patient Safety and Satisfaction With Fully Remote Management of Radiation Oncology Care
Source: JAMA Netw Open. 2024 Jun 12;7(6):e2416570. doi: 10.1001/jamanetworkopen.2024.16570 (PMC11170299; doi:10.1001/jamanetworkopen.2024.16570)
Supplement: Supplement 2. — Data Sharing Statement [file jamanetwopen-e2416570-s002.pdf]

Data Sharing Statement

Cuaron. Patient Safety and Satisfaction With Fully Remote Management of Radiation Oncology Care. *JAMA Netw Open*. Published June 12, 2024.
doi:10.1001/jamanetworkopen.2024.16570

Data
Data available: Yes
Data types: Deidentified participant data, Data dictionary
How to access data: cuaronj@mskcc.org
When available: With publication

Supporting Documents
Document types: None

Additional Information
Who can access the data: Researchers whose proposed use of the data has been approved
Types of analyses: Multi-institutional collaboration of similar topic
Mechanisms of data availability: After approval of appropriate proposal an with signed data access agreement.
